# Uridine prevents tamoxifen-induced liver lipid droplet accumulation

**DOI:** 10.1186/2050-6511-15-27

**Published:** 2014-05-23

**Authors:** Thuc T Le, Yasuyo Urasaki, Giuseppe Pizzorno

**Affiliations:** 1 Nevada Cancer Institute, One Breakthrough Way, Las Vegas, NV 89135, USA; 2 Desert Research Institute, 10530 Discovery Drive, Las Vegas, NV 89135, USA; 3 Roseman University of Health Sciences, 11 Sunset Way, Henderson NV 89014, USA

**Keywords:** Coherent anti-Stokes Raman scattering microscopy, Drug-induced fatty liver, Lipidomics, Membrane phospholipid, Mitochondrial respiration, Protein lysine acetylation, Pyrimidine, Tamoxifen, Triacylglyceride, Uridine phosphorylase

## Abstract

**Background:**

Tamoxifen, an agonist of estrogen receptor, is widely prescribed for the prevention and long-term treatment of breast cancer. A side effect of tamoxifen is fatty liver, which increases the risk for non-alcoholic fatty liver disease. Prevention of tamoxifen-induced fatty liver has the potential to improve the safety of long-term tamoxifen usage.

**Methods:**

Uridine, a pyrimidine nucleoside with reported protective effects against drug-induced fatty liver, was co-administered with tamoxifen in C57BL/6J mice. Liver lipid levels were evaluated with lipid visualization using coherent anti-Stokes Raman scatting (CARS) microscopy, biochemical assay measurement of triacylglyceride (TAG), and liquid chromatography coupled with mass spectrometry (LC-MS) measurement of membrane phospholipid. Blood TAG and cholesterol levels were measured. Mitochondrial respiration of primary hepatocytes in the presence of tamoxifen and/or uridine was evaluated by measuring oxygen consumption rate with an extracellular flux analyzer. Liver protein lysine acetylation profiles were evaluated with 1D and 2D Western blots. In addition, the relationship between endogenous uridine levels, fatty liver, and tamoxifen administration was evaluated in transgenic mice *UPase1*^−/−^and *UPase1*-TG.

**Results:**

Uridine co-administration prevented tamoxifen-induced liver lipid droplet accumulation in mice. The most prominent effect of uridine co-administration with tamoxifen was the stimulation of liver membrane phospholipid biosynthesis. Uridine had no protective effect against tamoxifen-induced impairment to mitochondrial respiration of primary hepatocytes or liver TAG and cholesterol export. Uridine had no effect on tamoxifen-induced changes to liver protein acetylation profile. Transgenic mice *UPase1*^−/−^with increased pyrimidine salvage activity were protected against tamoxifen-induced liver lipid droplet accumulation. In contrast, *UPase1*-TG mice with increased pyrimidine catabolism activity had intrinsic liver lipid droplet accumulation, which was aggravated following tamoxifen administration.

**Conclusion:**

Uridine co-administration was effective at preventing tamoxifen-induced liver lipid droplet accumulation. The ability of uridine to prevent tamoxifen-induced fatty liver appeared to depend on the pyrimidine salvage pathway, which promotes biosynthesis of membrane phospholipid.

## Background

Tamoxifen is an effective drug widely used for the treatment of estrogen receptor-positive breast cancer [1]. Women taking tamoxifen from 5 to 10 years exhibit reduced risks of breast cancer recurrence and mortality [2,3]. While generally well-tolerated, tamoxifen is known to induce fatty liver in 43% of women within the first 2 years of treatment [4-6]. Fatty liver is an established risk factor for non-alcoholic fatty liver disease (NAFLD) [7]. Prolonged tamoxifen treatment increases the risk of NAFLD, particularly in women with pre-existing metabolic condition [8].

The mechanism underlying tamoxifen-induced fatty liver is a topic of active investigation. Evidence from several independent research groups supports tamoxifen-induced impairment of mitochondrial fatty acid oxidation (FAO) as a primary cause of lipid accumulation in the liver [9-11]. Co-administration of tetradecylthioacetic acid, which improves mitochondrial and peroxisomal FAO, prevents tamoxifen-induced fatty liver [12]. Tamoxifen also inhibits hepatic triacylglyceride secretion leading to liver lipid accumulation [10,11]. Therapeutic intervention to prevent tamoxifen-induced fatty liver condition has the potential to improve the safety of long-term tamoxifen usage for breast cancer treatment.

**Figure 1:**
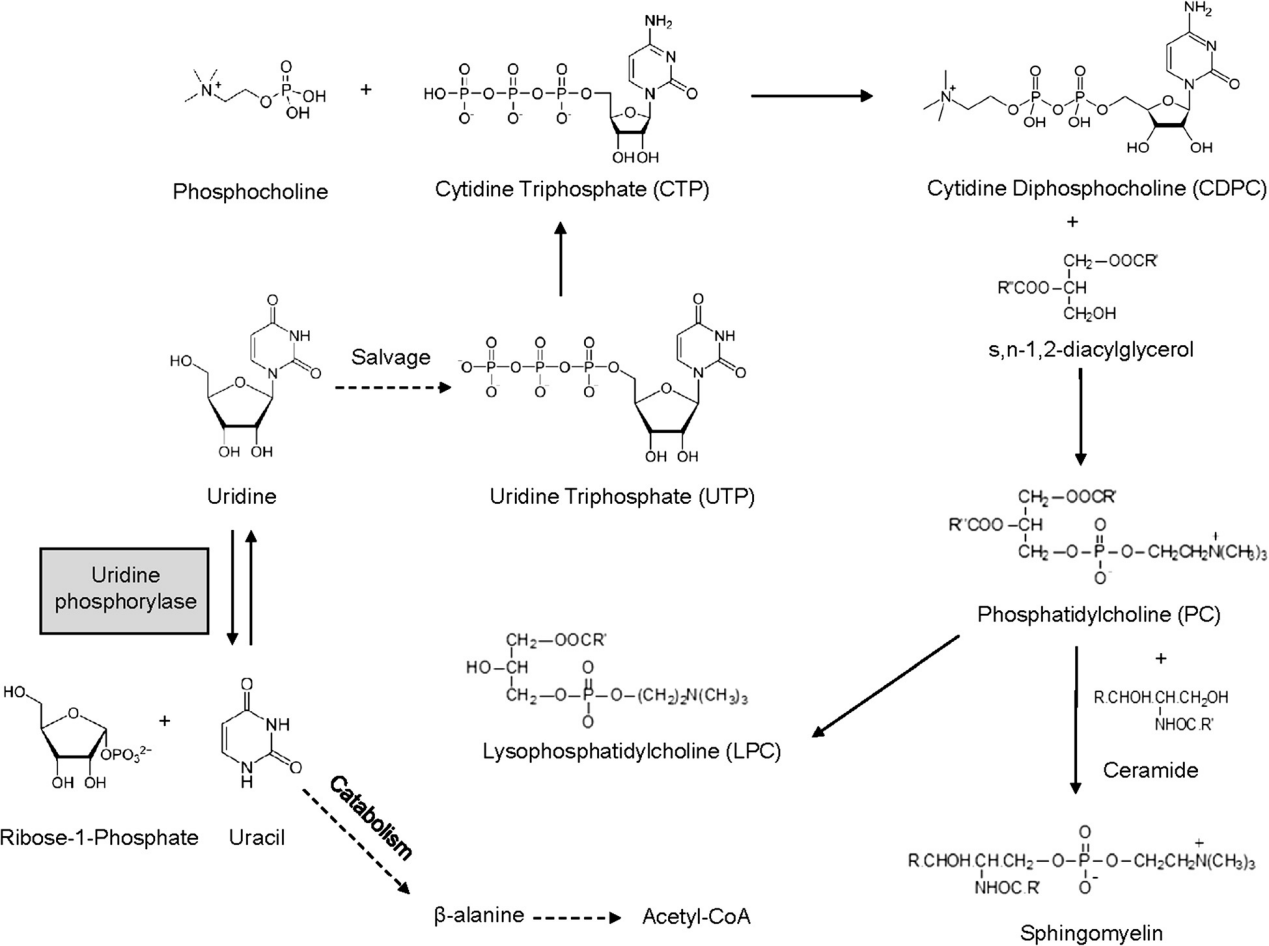
**Uridine salvage and membrane phospholipid biosynthesis.** Dashed arrows indicate multiple enzymatic reactions.

Uridine, a pyrimidine nucleoside, has been shown to prevent fatty liver condition induced by several drugs with unrelated therapeutic usages and acting mechanisms [13,14]. Uridine could be salvaged into pyrimidine nucleotides or catabolized into uracil and subsequently β-alanine and acetyl-CoA (Figure 1) [15]. Homeostatic regulation of uridine is controlled by uridine phosphorylase, an enzyme that catalyzes the reversible phosphorylitic conversion of uridine to uracil [16]. Genetic knock-out of uridine phosphorylase in *UPase1*^−/−^mice elevates tissues and plasma levels of uridine [17]; whereas, transgenic overexpression of uridine phosphorylase in *UPase1*-TG mice depletes tissues and plasma levels of uridine [18]. The liver is actively regulating plasma uridine level by continuously degrading plasma uridine and replacing it with *de novo* uridine synthesis [19]. The interaction between liver uridine homeostasis and lipid metabolism has been reported [18]. However, precise underlying mechanisms have not been determined. Consequently, therapeutic potential of uridine for treatment of fatty liver condition has not been realized.

In this study, we examine the effects of uridine co-administration with tamoxifen on liver lipid content in control C57BL/6J and transgenic *UPase1*^−/−^and *UPase1*-TG mice. Specifically, we examine the contribution of pyrimidine salvage and catabolism pathways to the biological activity of uridine. We aim to explore therapeutic potential of uridine for the prevention of drug-induced fatty liver and biological action of uridine on liver lipid metabolism.

## Methods

### Ethical statement

All animal studies were performed with the ethical approval of the Animal Care and Use Committees at Nevada Cancer Institute, Desert Research Institute, and Touro University Nevada. All experiments conducted on animals were in compliance with the guidelines of the U.S. Office of Laboratory Animal Welfare of the National Institutes of Health and the Public Health Service Policy on Humane Care and Use of Laboratory Animals.

### Experimental animals

Three mice strains were used, C57BL/6J or wildtype mice (Jackson Laboratories, Bar Harbor, ME), *UPase1*-TG mice with ubiquitous genetic knock-in of uridine phosphorylase 1 [18], and *UPase1*^−/−^mice with ubiquitous genetic knock-out of uridine phosphorylase 1 [17]. Transgenic mice described in this study have been deposited into the Mutant Mouse Regional Resource Centers supported by the National Institutes of Health. The MMRRC strains are now known as B6;129-*Upp1*^*tm1Gp*^/Mmucd (037119-UCD) for *UPase1*^−/−^mice and B6; FVB-*Gt (ROSA) 26Sor*^*tm1.1(CAG-Upp1)Gp*^/Mmucd (037120-UCD) for *UPase1*-TG mice. All mice used were female at 10–12 weeks of age with average bodyweight of approximately 20 grams.

### Study design

All mice were randomly divided into groups of 4 or 5 mice per cage and housed in a controlled environment with an average temperature of 22°C, a 12 hours of light and 12 hours of dark cycle, and with *ad libitum* access to food and water. For control C57BL/6J mice, 36 mice were randomly divided into 4 experimental groups of 9 mice per group: control diet (C57BL/6J), diet supplemented with uridine (C57BL/6J + U), diet supplemented with tamoxifen (C57BL/6J + Tmx), and diet supplemented with both tamoxifen and uridine (C57BL/6J + Tmx + U). For transgenic *UPase1*^−/−^and *UPase1*-TG mice, 18 mice per strain were randomly divided into the following 4 experimental groups of 9 mice per group: *UPase1*^−/−^mice on control diet (*UPase1*^−/−^), *UPase1*^−/−^mice on tamoxifen-supplemented diet, *UPase1*-TG mice on control diet (*UPase1*-TG), and *UPase1*-TG mice on tamoxifen-supplemented diet. In addition, 6 C57BL/6J mice were used for primary hepatocyte collection for bioenergetics experiments. The number of mice per experimental group was chosen to ensure that data obtained were statistically significant.

### Experimental procedures

Control mice were fed with PicoLab Mouse Diet ground pellets (Cat. No. 5058, LabDiet, Brentwood, MO) that provide 4.6 kcal/g and consist of 22% protein and 9% fat. The lipid composition includes cholesterol (200 ppm), linoleic acid (2.32%), linolenic acid (0.21%), arachidonic acid (0.02%), and omega-3 fatty acid (0.32%). The total saturated and monounsaturated fatty acids are 2.72% and 2.88%, respectively. For mice receiving uridine supplementation alone, uridine was thoroughly mixed with ground pellets with a dosage of 400 mg/kg/day. For mice receiving tamoxifen treatment alone, tamoxifen was thoroughly mixed with ground pellets with a dosage of 200 mg/kg/day. For mice receiving both uridine and tamoxifen, uridine and tamoxifen were thoroughly mixed with ground pellets with a dosage of 400 mg/kg/day and 200 mg/kg/day, respectively. Mice were placed on control or supplemented diets for 5 days prior to terminal liver and blood samples collection. All samples were collected in early mornings. Blood samples were collected via the tail veins while mice were under anesthesia with isoflurane. Liver tissues were collected following cardiac perfusion under deep anesthesia with isoflurane. Cardiac perfusion was necessary to ensure collection of pure liver tissues devoid of blood and plasma contaminants. Mice were anaesthetized and incisions were made from the abdomen up to the torso. Diaphragms were severed and 22 gauge needles were inserted into the left ventricles. Phosphate buffered saline (PBS) was used as the perfusate. Approximately 50–100 ml of PBS was flushed through each mouse from the left ventricle and exited through the incision made to the right atrium. Following the perfusion procedure, liver tissues were collected for immediate usage or frozen in liquid nitrogen for future usage.

### CARS imaging of liver tissues

A home-built CARS microscope was used to image lipid using CH_2_ vibrational frequency at 2851 cm^−1^ as described previously [20]. Approximately 31 frames were taken along the vertical axis at 1-micron increment for volumetric evaluation of liver lipid content. Liver lipid level was the square root of resonant CARS signal intensity, which is the difference between total CARS signal intensity and CARS signal intensity arising from cellular membrane and non-resonant signal [21-23]. Liver lipid level was normalized to 1 for control wildtype mice and respectively for other mice strains or treatment conditions. Quantitative analysis of liver lipid level was performed using the NIH ImageJ software. Liver was perfused with PBS prior to collection. Liver tissues were sliced into 200-micron thick sections, transferred into glass-bottom chambered slides, overlaid with 200 microliter of 1% agarose, and imaged with CARS microscopy. On average, 9 imaging volumes were analyzed with CARS microscopy per liver sample. The xyz dimensions of each analysis volume were 167 μm × 167 μm × 30 μm. Nine liver samples from nine mice were used for CARS imaging analysis per animal group.

### Biochemical measurement of liver triacylglyceride (TAG)

Liver samples of equal weight were used for chloroform/methanol total lipid extraction. TAG was determined using the commercial TAG quantitation kit (Cat. No. 10010303, Cayman Chemical, Ann Arbor, MI) according to manufacturer’s protocol and normalized with liver tissue weight. Nine liver samples from nine mice were used for biochemical TAG measurement per animal group.

### 1D Western blots

Total liver protein extracts were separated on 10% SDS-PAGE gels, transferred to nitrocellulose membranes, incubated first with primary antibodies against proteins of interest and then with secondary antibodies conjugated with horseradish peroxidase (Cat. No. 31460, Thermo Scientific, Rockford, IL). Membrane was developed with enhanced chemiluminescence reagents (Cat. No. 34075, Thermo Scientific), stripped, and re-incubated with antibodies against β-actin for evaluation of loading controls. Primary antibodies against acetylated lysine and β-actin were from Cell Signaling (Cat. No. 9441 & 4967, Danvers, MA).

### 2D Western blots

2D Western blots were performed by Kendrick Laboratories (Madison, WI). Approximately 500 μg of protein from each liver tissue was loaded per gel. Proteins were separated using isoelectric focusing (IEF) in the first dimension and SDS polyacrylamide gel electrophoresis (SDS-PAGE) in the second dimension. Primary and secondary antibodies were the same as in 1D Western blot. Molecular weight standards were: myosin (220,000), phosphorylase A (94,000), catalase (60,000), actin (43,000) carbonic anhydrase (29,000) and lysozyme (14,000) (Sigma Chemical Co., St. Louis, MO).

### Bioenergetics of primary hepatocytes

Immediately after isolation, primary hepatocytes were plated into 24-well plates at a density of 1 × 10^5^ cell per well. Plating media was consisted of DMEM with 25 mM glucose, 2 mM glutamine, 10% FBS, 0.1 mM sodium pyruvate, 1% Pen/Strep, and 1 mM HEPES at pH 7.4. At 4 hours after plating, primary hepatocytes were incubated for 24 hours with either uridine alone, tamoxifen alone, or a combination of tamoxifen and uridine depending on the treatment condition. The final concentration used for uridine was 100 μM and tamoxifen was 10 μM. At 90 minutes prior to assaying, plating media was replaced with Cellular Assay Solution consisting of DMEM, 25 mM glucose, 2 mM glutamine, 1 mM sodium pyruvate and adjusted to pH 7.2 with 25 mM of MOPS. Bioenergetics of primary hepatocytes were determined using the XF Cell Mito Stress Test Kit and a XF24-3 Analyzer (Seahorse Bioscience, North Billerica, MA) following manufacturer’s suggested protocols and published protocols [24]. Bioenergetics experiments were performed at the UCLA’s Cellular Bioenergetics Core Facilities. At least 24 repeated measurements were performed per experimental condition. Final concentrations of oligomycin, FCCP, rotenone, and myxothiazol were 1 μg/ml, 1 μM, 0.1 μM, and 2 μM, respectively. Oxygen consumption rates were reported as absolute values (pmol O_2_ consumed per minute) on a per-unit of protein basis, where average protein concentration per well was normalized to 1.

### Clinical blood lipid analysis

Analysis of blood lipid level (TAG, cholesterol, HDL, and LDL) were performed by Research Animal Diagnostic Laboratory (RADIL, Columbia, MO) on terminally collected blood samples of 6 mice per animal group. HDL and LDL were determined via direct measurement.

### Measurement of phospholipid with LC-MS

Total liver lipid extracts of 6 mice per animal group were sent to Kansas Lipidomics Research Center (Kansas State University, Manhattan, KS) for LC-MS analysis of phospholipid species. Concentrations of phospholipid are expressed as nmol per mg of dried liver lipid weight.

### Statistical analysis

Data were presented as average values ± standard deviations. Statistical analysis was performed using Excel’s paired Student’s t-test and analysis of variance (ANOVA) functions. Statistical significance was set at p ≤ 0.05.

## Results and discussion

**Figure 2:**
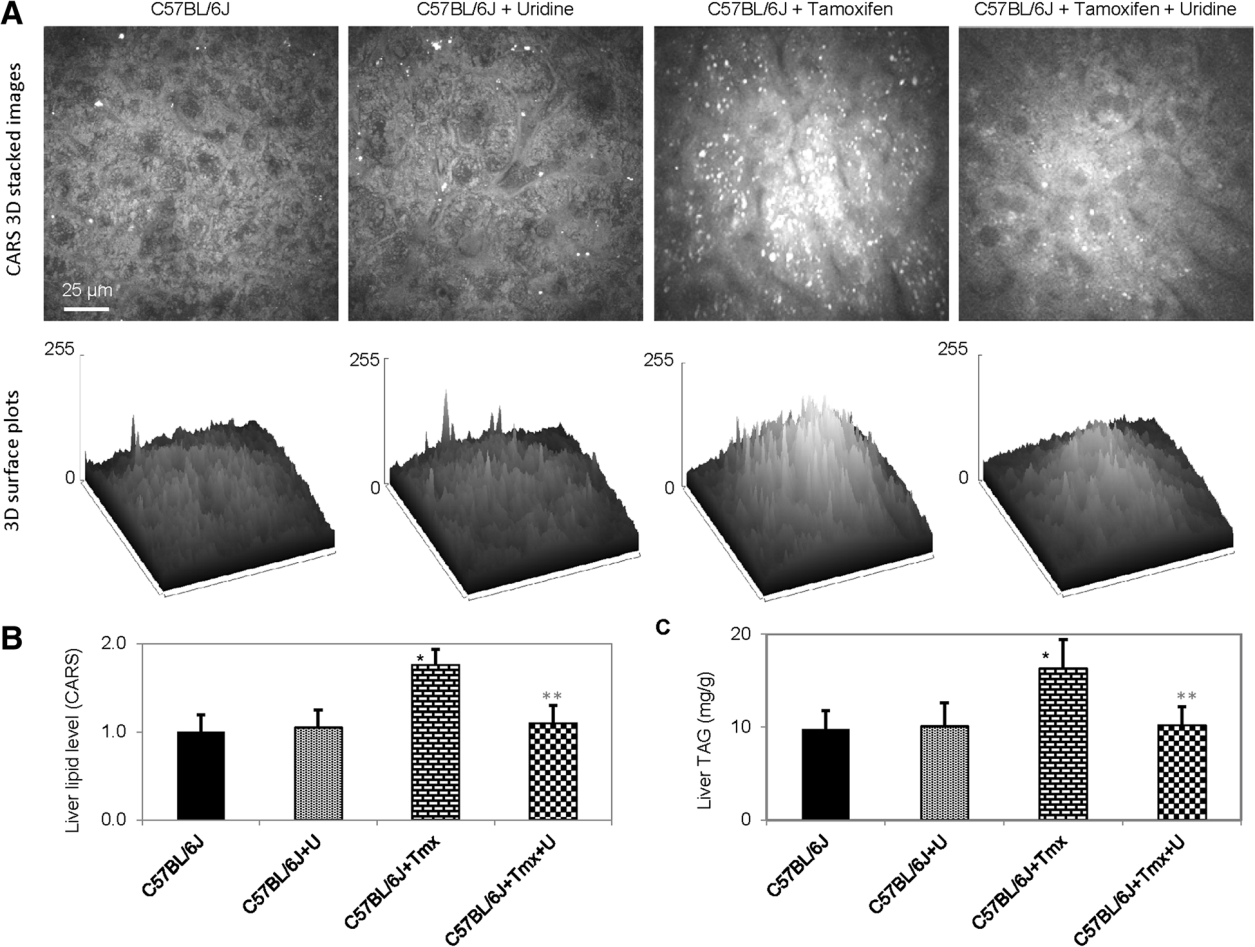
**Uridine prevents tamoxifen-induced fatty liver. (A)** CARS images (upper panels) and 3D surface plots of lipid distribution of C57BL/6J liver tissues as a function of uridine and/or tamoxifen treatment. **(B)** Liver lipid level determined with CARS image analysis. **(C)** Liver triacylglyceride (TAG) levels determined with biochemical assays. Error bars are standard deviation values across 9 mice analyzed per animal group. Single asterisk (black) indicates p-value < 0.05 versus untreated control mice. Double asterisks (gray) indicate p-value < 0.05 versus C57BL/6J mice treated with tamoxifen.

The effects of tamoxifen treatment on liver lipid content were evaluated in C57BL/6J mice. Daily dosage of 200 milligrams tamoxifen per kilogram bodyweight was chosen for mice to reproduce equivalent dosage administered in humans after adjusting for differences in energy metabolism and pharmacokinetics between species [25]. Following 5 days of tamoxifen treatment, mice lost up to 5% of bodyweight. Traditional histopathology analysis does not have sufficient sensitivity to analyze mild hepatic microvesicular steatosis associated with tamoxifen treatment for 5 days [11,21]. Therefore, collected liver tissues were examined with CARS microscopy, a highly sensitive method for lipid visualization [22,23,26], to evaluate liver lipid content. Liver tissues of untreated control mice and mice treated with uridine did not exhibit any intracellular lipid accumulation (Figure 2A). In contrast, liver tissues of mice treated with tamoxifen had significant intracellular lipid droplet accumulation, which could be classified as microvesicular steatosis [27]. Quantitative analysis of liver lipid level using CARS signal intensity revealed that tamoxifen treatment increased intracellular liver lipid level by 76% (Figure 2B). Surprisingly, uridine co-administration completely prevented tamoxifen-induced hepatic steatosis (Figure 2A,B). Data obtained with CARS microscopy were corroborated with biochemical measurements of liver triacylglyceride content (Figure 2C). Oil Red O histology was also performed on all liver tissue samples. However, ORO histology was unable to detect mild hepatic microvesicular steatosis associated with tamoxifen treatment (data not shown). The insensitivity of ORO histology to detect mild microvesicular steatosis had been described in the literature [21,28-30]. It is important to point out that while uridine co-administration completely suppressed tamoxifen-induced hepatic microvesicular steatosis, it had no impact on tamoxifen-induced weight loss in mice.

**Figure 3:**
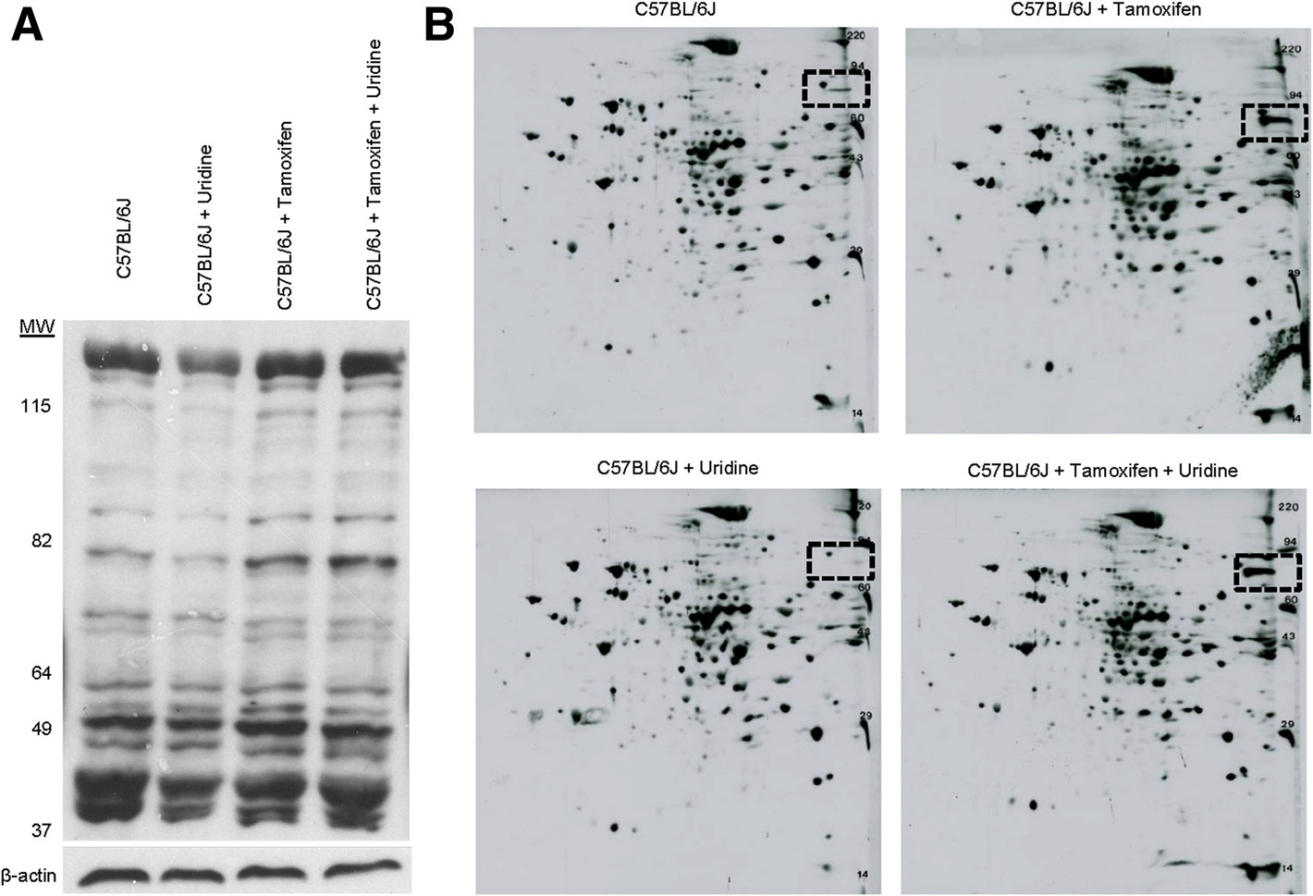
**Effects of uridine and tamoxifen on liver protein acetylation profile. (A)** 1D Western blot analysis of liver protein lysine acetylation profile. β-actin serves as a loading control. **(B)** 2D Western blot analysis of liver protein lysine acetylation profile. Box with dashed black lines highlights the locations of a protein band at 80 kD whose acetylation level increases with tamoxifen treatment.

Uridine has the ability to modulate liver protein acetylation profile [14,18]. Uridine catabolism produces acetyl-CoA, which is a donor substrate for protein acetylation [18]. Uridine supplementation also elevates liver NAD^+^/NADH ratio, which alters the activity of NAD^+^-dependent deacetylases [18]. Liver protein acetylation is highly correlated to energy metabolism [31,32]. To determine whether uridine prevented tamoxifen-induced fatty liver by modulating protein acetylation profile, 1D Western blots using antibodies against acetylated lysine residues were performed on total liver extracts (Figure 3A). Tamoxifen treatment increased acetylation of a protein band with molecular weight of ~80 kD. However, uridine co-administration with tamoxifen had no effect on the acetylation state of this protein band.Next, 2D Western blots were employed for high resolution evaluation of liver protein acetylation profiles (Figure 3B). Consistent with 1D Western blots, 2D Western blots revealed that tamoxifen treatment induced acetylation of a protein spot with molecular weight of 80 kD (Figure 3B, box with dashed line). Also consistent with 1D Western blot, uridine co-administration with tamoxifen could not prevent the effect of tamoxifen-induced hyper-acetylation of this 80 kD protein spot. Overall, when uridine was co-administered with tamoxifen, it had no impact on liver protein acetylation profile. Therefore, it was unlikely that uridine prevented tamoxifen-induced fatty liver by modulating liver protein acetylation profiles.

**Figure 4:**
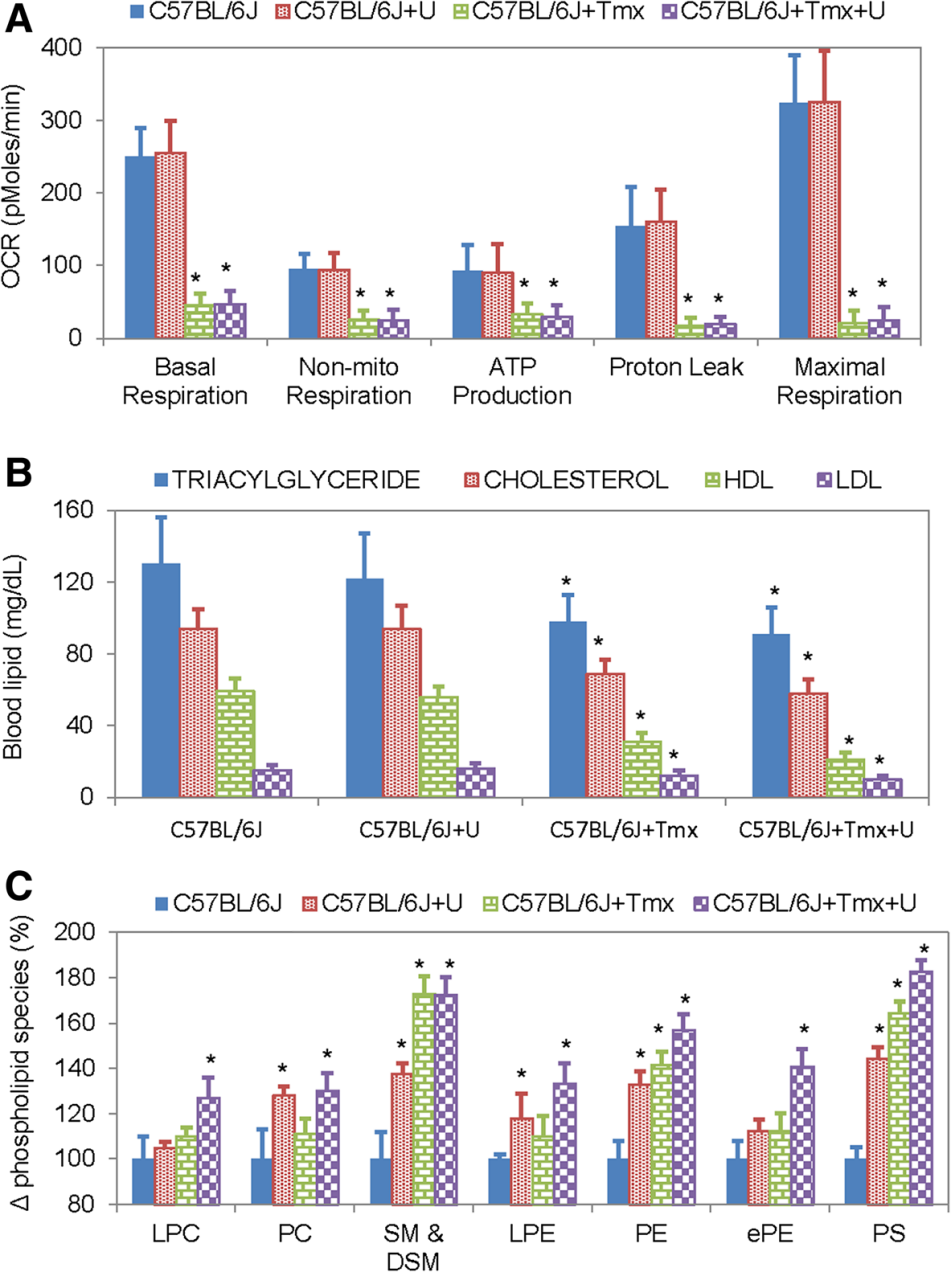
**Effects of uridine and tamoxifen on hepatocyte mitochondrial respiration, blood lipid level, and liver phospholipid level. (A)** Oxygen consumption rate (OCR) of primary hepatocytes measured with an Extracellular Flux Analyzer. Error bars are standard deviation values across 24 repeated measurements per experimental condition. **(B)** Blood levels of triacylglyceride, cholesterol, high-density lipoprotein (HDL), and low-density lipoprotein (LDL) determined via direct measurements. **(C)** Changes in selective phospholipid species as a function of uridine and/or tamoxifen treatment. Lysophosphotidylcholine (LPC); phosphotidylcholine (PC); sphingomyelin (SM) & dihydrosphingomyelin (DSM); lysophosphoethanolamine (LPE); phosphoethanolamine (PE); ether-linked phosphoethanoamine (ePE); phosphatidylserine (PS). Error bars are standard deviation values across 6 mice analyzed per animal group. Asterisks indicate p-value < 0.05 versus untreated control mice.

Tamoxifen treatment is associated with impaired mitochondrial respiration [9,11]. To determine whether uridine co-administration could prevent the inhibitory effects of tamoxifen on mitochondrial respiration, primary hepatocyte cell cultures were employed. An Extracellular Flux Analyzer was employed to measure 5 key parameters of cellular bioenergetics: basal respiration, non-mitochondrial respiration, ATP production, proton leak, and maximal respiration using a previously described protocol [24]. Consistent with the literature, tamoxifen treatment severely reduced oxygen consumption rates in primary hepatocytes for all parameters evaluated (Figure 4A). Uridine administration by itself had no effect on mitochondrial respiration of primary hepatocytes. Surprisingly, uridine co-administration could not prevent tamoxifen-induced impairment to mitochondrial respiration in primary hepatocytes. Thus, it was unlikely that uridine co-administration prevented fatty liver by restoring mitochondrial function impaired by tamoxifen.

Tamoxifen treatment is associated with reduced liver secretion of TAG and cholesterol [10,11]. To determine if uridine co-administration with tamoxifen could improve liver secretion of TAG and cholesterol, blood lipid profiles were evaluated (Figure 4B). Consistent with the literature, tamoxifen treatment caused reduction of blood TAG and cholesterol levels. Uridine treatment by itself had no effect on blood TAG and cholesterol levels. Neither could uridine co-administration prevent tamoxifen-induced reduction of blood TAG and cholesterol levels. Thus, there was no evidence to support a role of uridine in reversing tamoxifen-induced suppression of liver secretion of TAG and cholesterol.

Salvage of uridine to cytidine triphosphate promotes phospholipid biosynthesis in the presence of phosphocholine and diacylglycerol [15,33]. To determine if there was a role for uridine salvage in the prevention of tamoxifen-induced fatty liver, lipidomics profiling of liver phospholipid with LC-MS was carried out (Figure 4C, Table 1). When administered individually, both uridine and tamoxifen increased the levels of many liver phospholipid species. When administered together, uridine and tamoxifen further increased the levels of many liver phospholipid species. Among the most affected phospholipid species were sphingomyelin, phosphatidylserine, and phosphoethanolamine, where uridine co-administration with tamoxifen increased their levels by as much as 80%. The lipidomics data suggested that uridine could prevent tamoxifen-induced fatty liver by promoting membrane phospholipid biosynthesis.

**Table 1:** Liver phospholipid species quantified with LC-MS

**Phospholipid species (nmol/mg)**	**C57BL/6J**	**C57BL/6J + U**	**C57BL/6J + Tmx**	**C57BL/6J + Tmx + U**
Lysophosphotidylcholine	6.7 ± 0.7	7.1 ± 0.3	7.4 ± 0.4	8.6 ± 0.8*
Phosphotidylcholine	380.5 ± 51	486.8 ± 20*	421.9 ± 30.6	495.1 ± 39.6*
Sphingomyelin & dihydrosphingomyelin	29.1 ± 3.7	40.0 ± 2.8*	50.3 ± 3.9*	50.2 ± 3.7*
Ether-linked phosphotidylcholine	18.9 ± 2.2	18.5 ± 0.6	21.2 ± 1.4	22.5 ± 2.1
Lysophosphoethanolamine	1.9 ± 0.1	2.3 ± 0.26	2.1 ± 0.2	2.6 ± 2.1
Phosphoethanolamine	105.5 ± 8.9	140.1 ± 8.8*	149.2 ± 8.3*	165.6 ± 8.9*
Phosphoethanoamine-ceramide	0.01 ± 0.01	0.01 ± 0.01	0.016 ± 0.005	0.027 ± 0.013
Ether-linked Phosphoethanoamine	4.0 ± 0.4	4.5 ± 0.3	4.5 ± 0.3	5.6 ± 0.4*
Phosphatidylinositol	51.2 ± 6.9	60.0 ± 7.1	46.9 ± 3.7	58.4 ± 6.2
Phosphatidylserine	15.4 ± 0.8	22.3 ± 1.1*	25.4 ± 1.3*	28.2 ± 1.3*
Ether-linked phosphatidylserine	0.2 ± 0.03	0.3 ± 0.03*	0.36 ± 0.017*	0.4 ± 0.04*
Phosphatidic acid	19.9 ± 2.3	23.2 ± 2.4	15.2 ± 1.7	21.2 ± 1.4
Phosphatidylglycerol	27.2 ± 3.1	29.9 ± 2.7	33.0 ± 2.1	29.7 ± 2.2

Asterisks indicate p-value <0.05 versus untreated control.

**Figure 5:**
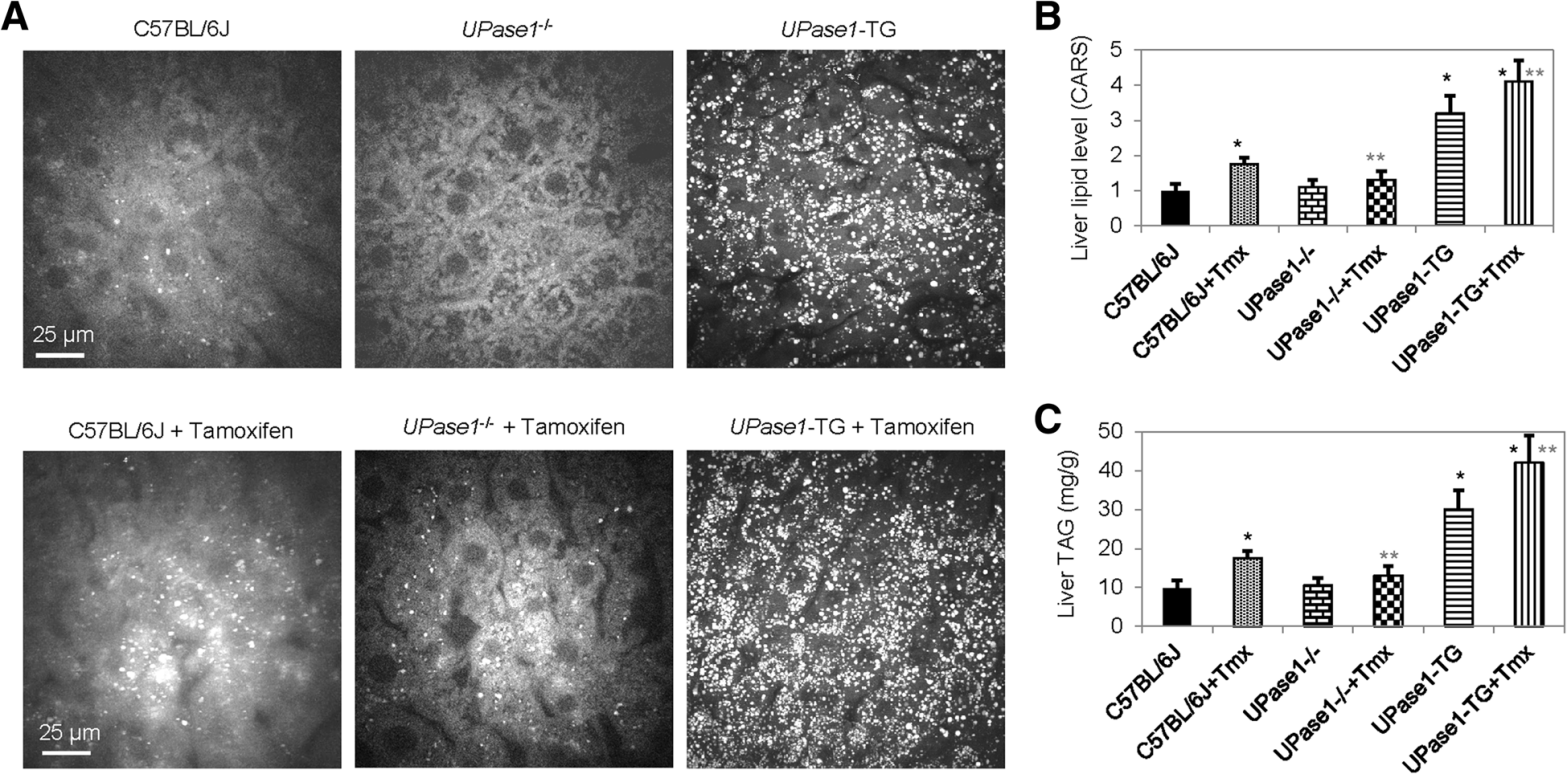
**Increased uridine salvage protects liver against tamoxifen-induced lipid accumulation. (A)** CARS images of liver tissues of wildtype C57BL/6J and transgenic *UPase1*^−/−^and *UPase1*-TG mice as a function of tamoxifen treatment. **(B)** Liver lipid level determined with CARS image analysis. **(C)** Liver triacylglyceride (TAG) levels determined with biochemical assays. Error bars are standard deviation values across 9 mice analyzed per animal group. Single asterisk (black) indicates p-value < 0.05 versus untreated control mice. Double asterisks (gray) indicate p-value < 0.05 versus C57BL/6J mice treated with tamoxifen.

To further evaluate the relationship between pyrimidine salvage pathway and the prevention of tamoxifen-induced fatty liver, *UPase1*^−/−^and *UPase1*-TG mice were employed. *UPase1*^−/−^mice have elevated liver and circulating uridine concentration due to genetic knock-out of a gene encoding for uridine phosphorylase 1, an enzyme that catalyzes uridine catabolism [17]. On average, *UPase1*^−/−^mice have liver and plasma concentration of 42.8 μM and 7.2 μM, respectively; whereas, C57BL/6J mice have liver and plasma concentration of 6.8 μM and 1.5 μM, respectively [34]. For *UPase1*^−/−^strain, liver tissues of untreated control mice were devoid of intracellular lipid droplet (Figure 5A-C). Tamoxifen treatment of *UPase1*^−/−^mice did not lead to intracellular lipid droplet accumulation in the liver tissues. On the other hand, *UPase1*-TG mice have depleted liver and circulating uridine concentration due to genetic knock-in of a gene encoding for for uridine phosphorylase 1 [18]. On average, *UPase1*-TG mice have liver and plasma uridine concentration of 0.5 μM and 0.08 μM, respectively. For *UPase1*-TG strain, liver tissues of untreated control mice were already exhibiting microvesicular steatosis (Figure 5A-C). Tamoxifen treatment of *UPase1*-TG mice increased liver lipid content by approximately 20% compared to untreated control *UPase1*-TG mice. Hence, *UPase1*^−/−^mice, which had increased pyrimidine salvage activity [17], were protected against tamoxifen-induced fatty liver. In contrast, *UPase1*-TG mice, which had increased uridine catabolism activity [18], were susceptible to further liver lipid accumulation following tamoxifen treatment.

## Conclusions

In this study, we report that uridine co-administration is effective at completely preventing intracellular lipid droplet accumulation in the liver tissues of mice treated with tamoxifen. To examine the roles of uridine in the prevention of tamoxifen-induced fatty liver, several aspects of liver energy metabolism perturbed by tamoxifen were evaluated. Tamoxifen administration was associated with an increased in acetylation of a protein band at 80 kD and impaired mitochondrial respiration; however, both of these tamoxifen-induced effects could not be reversed by uridine co-administration. Neither could uridine prevent tamoxifen-induced reduction in blood TAG and cholesterol levels. Surprisingly, both uridine and tamoxifen when administered alone and together increased membrane phospholipid biosynthesis. The synthesis of phosphatidylcholine (PC), the key component of phospholipid, was dependent on the availability of diacylglycerol (DAG) and cytidine diphosphocholine (CDPC) (Figure 1) [33]. It is possible that tamoxifen-induced lipid accumulation in liver tissues made TAG and DAG to be readily available for PC synthesis. Uridine salvage into CTP promoted CDPC synthesis, which together with DAG availability stimulated PC synthesis. Transgenic mice *UPase1*^−/−^with increased uridine salvage into CTP were protected against tamoxifen-induced fatty liver. In contrast, *UPase1*-TG mice with overt catabolism of uridine had intrinsic fatty liver phenotype, which was aggravated following tamoxifen treatment. In summary, uridine co-administration was able to prevent tamoxifen-induced intracellular lipid droplet accumulation, but not able to prevent other side effects associated with tamoxifen treatment such as impaired mitochondrial respiration and reduced TAG and cholesterol export. A plausible means that uridine prevented tamoxifen-induced fatty liver was via the pyrimidine salvage pathway, which channeled neutral lipid into phospholipid biosynthesis and reduced cytoplasmic lipid accumulation.

A previous study on the anti-proliferative effect of tamoxifen in human MCF-7 breast cancer cells proposed that tamoxifen prevented DNA synthesis by blocking uridine transport, thus, inhibiting the pyrimidine salvage pathway [35]. In our study, C57BL/6J mice with dietary uridine supplementation or *UPase1*^−/−^mice with elevated endogenous uridine levels both had enhanced pyrimidine salvage activity [17]. Both mice strains were resistant to tamoxifen-induced fatty liver; however, they exhibited weight loss following tamoxifen treatment. In addition, uridine supplementation in primary hepatocyte cultures could not prevent tamoxifen-induced impairment to mitochondrial respiration. Our observation indicates that tamoxifen exert inhibitory effects beyond the pyrimidine salvage pathway. Indeed, tamoxifen has been shown to directly intercalate mitochondrial DNA (mtDNA) and impair mtDNA synthesis and mitochondrial respiration [11]. It is unlikely that uridine can prevent the interaction of cationic tamoxifen with mtDNA, hence, its inability to suppress the inhibitory effects of tamoxifen on mitochondrial function.

The balance between purine and pyrimidine nucleotides is critical for the maintenance of genomic stability and regulation. A surge in uridine concentration subsequently leads to a rise in pyrimidine nucleotides and perturbs the balance of the nucleotide pool [15]. Therefore, an adaptive mechanism must be in place to cope with excessive uridine concentration. In rodents, most tissues rely on the plasma for uridine supply [36]. The circulating uridine concentration is tightly regulated by the liver, where plasma uridine is cleared in a single pass and replaced with newly synthesized uridine [19]. Hence, the liver effectively serves as a regulator of uridine homeostasis. Rapid clearance of uridine in the liver involves both uridine salvage and catabolism, where uridine metabolites affect other cellular processes in a non-specific manner. Multi-targeted effects are evident by the ability of uridine to prevent fatty liver caused by different drugs with vastly different acting mechanisms [13,14]. Multi-targeted effects pose a challenge for precise therapeutic targeting using uridine. However, uridine homeostasis is regulated by uridine phosphorylase [16]. The enzymatic activity of uridine phosphorylase has been modulated by pharmaceutical compounds to prevent toxicity associated with 5-fluorouracil treatment of cancer [37]. Modulation of uridine phosphorylase enzymatic activity is a possible means to achieve precise therapeutic targeting of uridine for the prevention of drug-induced fatty liver.

## Abbreviations

CARS: Coherent anti-Stokes Raman scattering; CDPC: Cytidine diphosphocholine; DAG: Diacylglycerol; HDL: High-density lipoprotein; LC-MS: Liquid chromatography coupled with mass spectrometry; LDL: Low-density lipoprotein; OCR: Oxygen consumption rate; PC: Phosphatidylcholine; TAG: Triacylglyceride.

## Competing interests

The authors declare that they have no competing interest.

## Authors’ contribution

TTL and GP designed experiments. TTL and GP contributed reagents, samples, and analytical tools. TTL and YU performed experiments and analyzed data. TTL prepared the manuscript. All authors read and approved final manuscript.

## Pre-publication history

The pre-publication history for this paper can be accessed here:

http://www.biomedcentral.com/2050-6511/15/27/prepub
